# Research on Helical Electrode Electrochemical Drilling Assisted by Anode Vibration for Jet Micro-Hole Arrays on Tube Walls

**DOI:** 10.3390/mi16010086

**Published:** 2025-01-13

**Authors:** Tao Yang, Yikai Xiao, Yusen Hang, Xiujuan Wu, Weijing Kong

**Affiliations:** 1 College of Mechanical Engineering, Nanjing Vocational University of Industry Technology, Nanjing 210023, China; xyk13770725152@163.com (Y.X.); 2023101416@niit.edu.cn (Y.H.); wuxj@niit.edu.cn (X.W.); kongwj@nuaa.edu.cn (W.K.); 2 Jiangsu Key Laboratory of Precision and Micro-Manufacturing Technology, Nanjing University of Aeronautics and Astronautics, Nanjing 210016, China; 3 Precision Manufacturing Engineering and Technology Research Center of Jiangsu Province, Nanjing Vocational University of Industry Technology, Nanjing 210023, China

**Keywords:** jet micro-hole arrays, electrochemical drilling, helical electrode, anode vibration

## Abstract

The electrochemical cutting technique, utilizing electrolyte flushing through micro-hole arrays in the radial direction of a tube electrode, offers the potential for cost-effective and high-surface-integrity machining of large-thickness, straight-surface structures of difficult-to-cut materials. However, fabricating the array of jet micro-holes on the tube electrode sidewall remains a significant challenge, limiting the broader application of this technology. To enhance the efficiency and quality of machining these jet micro-holes on the tube sidewall, a helical electrode electrochemical drilling method assisted by anode vibration has been proposed. The influence of parameters, such as the rotational direction and speed of the helical electrode, as well as the vibration amplitude and frequency of the workpiece, on the machining results was investigated using fluid field simulation and machining experiments. It was found that these auxiliary movements could facilitate the renewal of electrolytes within the machining gap, thereby enhancing the efficiency and quality of electrochemical drilling. Using the optimized machining parameters, an array of 10 jet micro-holes with a diameter of 200 μm was machined on the metal tube sidewall. Electrochemical cutting with radial electrolyte flushing tests were then performed through these micro-holes.

## 1. Introduction

Electrochemical cutting is a machining process based on the principle of electrochemical anodic dissolution, wherein a wire or rod tool electrode dissolves and removes localized material from the workpiece to complete the cut [1]. As a form of electrochemical machining, it offers high precision, excellent surface quality, tool electrode wear resistance, and no machining stress [2]. It is widely used for cutting and fabricating small precision components [3]. Furthermore, research is actively exploring its use for machining large-thickness ruled surface components, such as aviation turbine disk mortise structures, while maintaining high surface integrity [4,5].

A key limiting factor in electrochemical cutting is the restricted mass transport within the machining gap, which hampers both machining efficiency and quality [6]. To overcome this, a radial electrolyte flushing electrochemical cutting method using a tube electrode with an array of holes has been proposed. This method replaces traditional wire or rod electrodes with a metal tube featuring an array of jet holes in its sidewall. The electrolyte is injected rapidly through these arrayed jet holes into the machining gap, significantly improving mass transport and enhancing cutting efficiency [7]. The structure of the arrayed jet micro-holes on the tube sidewall plays a crucial role in the overall machining process [8], making their precise fabrication essential for the success of this technique [9].

Several methods exist for machining micro-holes, including micro-drilling, laser drilling, electrical discharge drilling, and electrochemical drilling. Micro-drilling removes material through the shear force generated by a micro drill, offering high machining efficiency [10]. However, drilling arrayed jet micro-holes on a tube wall is challenging due to tool inclination and potential breakage, especially in the early stages. Additionally, chips may enter the metal cavity, and burrs can form at the edges of the holes, interfering with subsequent electrolyte injection [11]. Laser drilling, which removes material through the localized melting and vaporization induced by high-energy laser beams, avoids these issues, as it does not require traditional drilling tools, thus eliminating tool wear. This non-contact process also avoids machining stresses, achieving high precision and efficiency for a variety of materials [12]. Similarly, electrical discharge drilling uses spark thermal energy to remove material, offering high efficiency and no contact between the tool and workpiece. It can also break through the material’s strength and hardness limits [13]. However, both laser and electrical discharge drilling introduce thermal defects, such as deformation, heat-affected zones, and recast layers, which may compromise the integrity of the drilled micro-holes and affect the tube’s subsequent performance [14].

Electrochemical drilling is a machining technique that removes material from a workpiece via electrochemical anode dissolution. In this process, localized material is dissolved in ionic form, ensuring high machining precision and a surface free from defects like burrs, recast layers, and heat-affected zones. Since the tool electrode does not physically contact the workpiece, processing stress is eliminated. Only hydrogen evolution occurs at the tool electrode surface, preventing tool wear [15]. This method is particularly advantageous for the high-quality manufacturing of jet micro-holes in tube walls [16]. For instance, micro-holes with a diameter of approximately 400 µm have been successfully machined in a 4 mm thick superalloy plate [17]. The use of a helical drill as the tool electrode significantly enhances both machining efficiency and micro-hole precision [18,19]. Liu et al. achieved a non-tapered micro-hole with a diameter of 186 µm in a 500 µm thick GH4169 plate by using a rotating helical electrode and ultra-short voltage pulses [20]. However, the removal efficiency of electrolytic products within the end-face machining gap between the end face of the helical electrode and the bottom of the hole still needs to be improved. To address this, Wang et al. utilized ultrasound to cause high-frequency vibration of the tool electrode, thereby facilitating the removal of products within the end-face machining gap. Ultimately, they were able to machine a deep hole with a depth of 5.4 mm and a depth-to-diameter ratio of 12.3 [21]. In this method, an excessively high ultrasonic vibration frequency can easily impact machining accuracy.

To improve the efficiency and quality of jet micro-hole processing on tube walls, we propose a helical electrode electrochemical drilling method assisted by anode vibration. The electrolyte within the machining gap is agitated by the high-speed rotational movement of the helical electrode, while the electrolyte in the end-face machining gap is intermittently squeezed by the vertical vibration of the anode. This promotes the flow of the electrolyte, accelerates the removal of electrolytic products and the renewal of the electrolyte, and consequently enhances machining efficiency and quality. Simulations and experiments were conducted to investigate the effects of the helical electrode’s rotational movement and the anode’s vibrational movement, and optimal machining parameters were selected. An array of jet micro-holes was machined on the sidewall of a metal tube, and a radial electrolyte flushing electrochemical cutting experiment was conducted using this tube with jet micro-holes as the electrode.

## 2. Machining Principle and Simulation Analysis

### 2.1. Machining Principle

**Figure 1:**
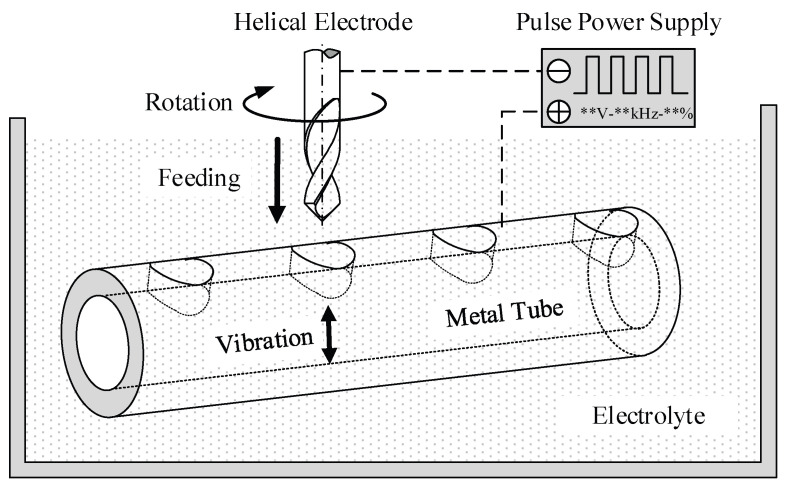
Principle schematic diagram of helical electrode electrochemical drilling assisted by anode vibration. ** is the specific electrical parameter value during processing.

Figure 1 illustrates the principle of helical electrode electrochemical drilling assisted by anode vibration on a metal tube sidewall. A tungsten steel alloy twist micro-drill is chosen as the helical electrode, and a stainless-steel 304 tube serves as the workpiece immersed in the electrolyte. The helical electrode is connected to the negative terminal, while the metal tube is connected to the positive terminal of the pulse power supply. Under the applied pulse voltage, an electrochemical reaction occurs between the tube wall and the helical electrode, resulting in the dissolution of localized material from the tube. As the helical electrode feeds, a micro-hole structure is formed on the tube sidewall. The high-speed rotation of the electrode stirs the electrolyte, promoting its renewal within the machining gap. The piezoelectric ceramic unit drives the tube to vibrate vertically, further enhancing the electrolyte flow and improving both machining stability and efficiency.

### 2.2. Simulation Analysis

**Figure 2:**
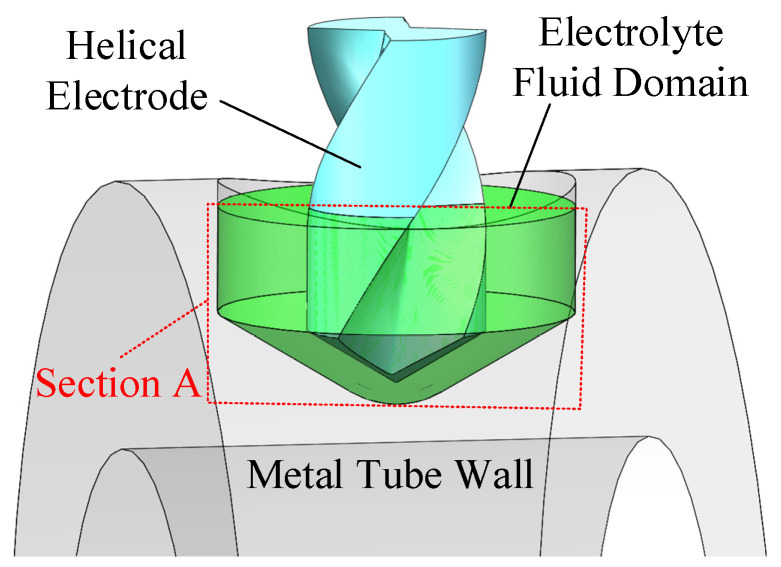
Simulation model of the electrolyte flow field.

A simulation study was conducted to examine the impact of the helical electrode’s rotational motion and the workpiece’s vertical vibration on the machining process, particularly the electrolyte flow field within the machining gap. Figure 2 shows the simulation model, with parameters detailed in Table 1. To clarify the electrolyte flow dynamics, a vertical section (A) was chosen as a reference.

**Figure 3:**
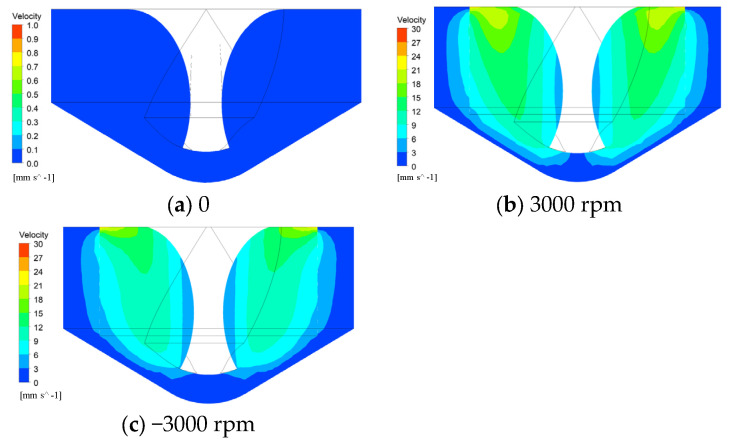
Distribution contour of electrolyte flow velocity within the machining gap during rotation of the helical electrode.

During the simulation process, the rotational speed of the helical electrode was set to 0 and 3000 rpm, respectively, to compare and analyze the impact of the helical electrode’s rotation on the electrolyte flow field. Additionally, the rotational speed of the helical electrode was set to −3000 rpm and 3000 rpm to compare and analyze the influence of the helical electrode’s rotational direction on the electrolyte flow field. Figure 3 presents the velocity contour of the electrolyte flow during the high-speed rotation of the helical electrode. Without rotation, the electrolyte remains stationary in the machining gap. However, at 3000 rpm, the helical electrode stirs the electrolyte, creating a localized flow that aids in the removal of electrolytic products and the renewal of the electrolyte. Furthermore, the velocity distribution of the electrolyte remains the same regardless of the direction of rotation. However, the electrolyte remains stagnant between the bottom surface of the tool and the bottom surface of the hole (the end-face machining gap). To address this, the application of workpiece vibration is proposed, aiming to drive the electrolyte flow by altering the size of the machining gap.

**Figure 4:**
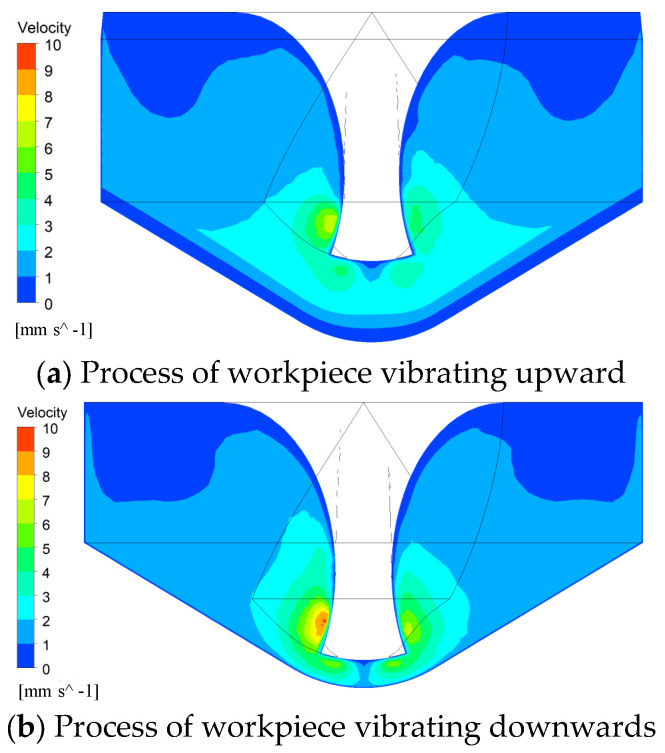
Distribution contour of electrolyte flow velocity within the machining gap during workpiece vibration.

During the up and down vibration of the workpiece, when the workpiece moves upwards, the end-face machining gap narrows, exerting a compressive force on the electrolyte. Conversely, when the workpiece moves downward, the end-face machining gap widens, creating a suction effect on the electrolyte. Therefore, the simulation focused on exploring both the upward and downward vibration processes of the workpiece. The amplitude and frequency of the vibration were set to 10 µm and 100 Hz, respectively. Figure 4 demonstrates the effect of workpiece vibration on the electrolyte flow field. Vertical movement of the workpiece causes periodic expansion and contraction of the machining gap, generating a pumping and squeezing effect that drives the electrolyte flow. This accelerates the removal of electrolytic products and promotes electrolyte renewal.

## 3. Experimental Details

**Figure 5:**
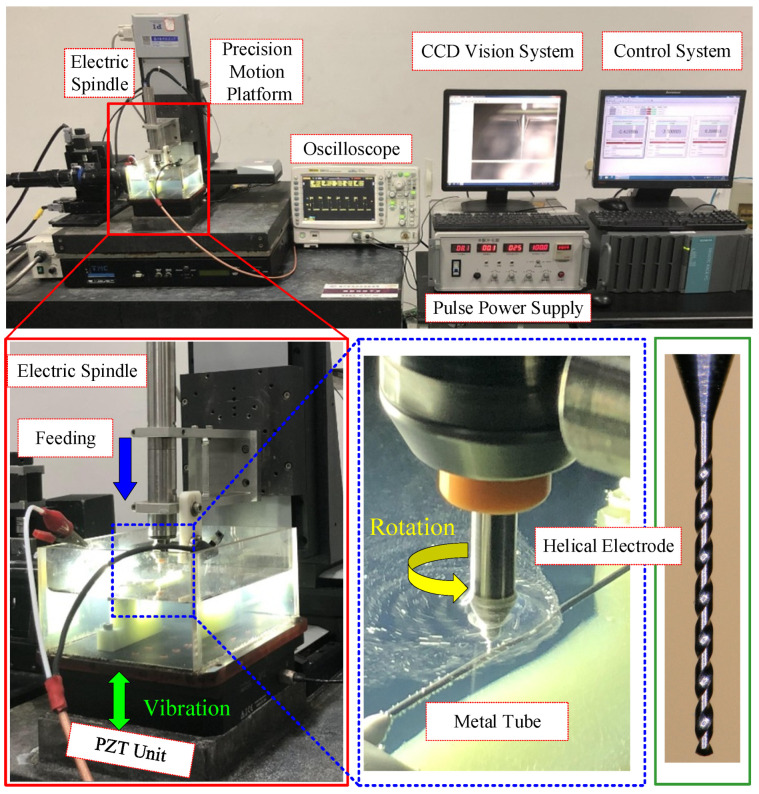
Experimental setup of helical electrode electrochemical drilling assisted by anode vibration.

Figure 5 shows the experimental setup for helical electrode electrochemical drilling assisted by anode vibration. The system includes a motion control system, an X/Y/Z precision motion platform (M511.DG, PI, Karlsruhe, Germany), an electric spindle (NR-2551, NSK, Tokyo, Japan), a piezoelectric ceramic unit (P158.ZCD, PI, Karlsruhe, Germany), a pulse power supply (YS9000D, Yisheng, Shanghai, China), an oscilloscope (DS6104, RIGOL, Suzhou, China), a Charge-Coupled Device (CCD) vision system, and a dedicated fixture and electrolyte tank. The stainless-steel tube is secured in the fixture within the electrolyte tank, where it is immersed in the electrolyte. The helical electrode, mounted on the electric spindle, undergoes high-speed rotation. The X/Y/Z precision motion platform controls the electrode’s feed motion, while the piezoelectric ceramic units drive the tube’s vertical vibration. The pulse power supply operates at a frequency of 1–100 kHz, ensuring both precision and efficiency. The positive and negative terminals of the power supply are connected to the tube and the helical electrode, respectively. The oscilloscope monitors the voltage and current in the circuit while the CCD vision system observes and records the machining process. Specific experimental parameters are listed in Table 2.

During electrochemical drilling, the helical electrode’s rotational motion and the workpiece’s vertical vibration influence electrolyte flow within the machining gap, affecting bubble removal and the discharge of insoluble electrolytic products. As outlined in Equation (1), variations in product removal efficiency alter the volume fraction of electrolytic products in the electrolyte, which in turn impacts its conductivity. According to Equation (2), when the helical electrode’s feed rate is constant, changes in electrolyte conductivity affect the end-face machining gap. In a balanced machining state, the relationship between the side-face and end-face machining gaps is defined by Equation (3). Equation (4) correlates the drilled hole size with the side-face machining gap. In this experiment, a constant feed rate is used, and the effects of the helical electrode’s rotational movement and the workpiece’s vertical vibration on machining results are analyzed by comparing hole diameters produced under varying auxiliary motion parameters.

(1) $$\kappa =\kappa_{0}\frac{2(1-\beta )}{2+\beta }$$

(2) $$\Delta_{b}=\frac{\eta \omega \kappa U_{R}}{v_{b}}$$

(3) $$\Delta_{s}≅\Delta_{b}\sqrt{1+\frac{2D_{TE}}{\Delta_{b}}}$$

(4) $$D_{H}=D_{TE}+2\Delta_{s}$$

Among them, κ is the conductivity of the electrolyte, $\kappa_{0}$ is the initial conductivity of the electrolyte, β is the content of electrolytic products in the electrolyte, $\Delta_{b}$ is the end-face machining gap, η is the current efficiency, ω is the volumetric electrochemical equivalent, $U_{R}$ is the voltage between the two electrodes, $v_{b}$ is the feed rate of the tool electrode, $\Delta_{s}$ is the side machining gap, $D_{\mathrm{TE}}$ is the diameter of the tool electrode, and $D_{H}$ is the diameter of the hole.

Ten holes are drilled for each set of machining parameters. After drilling, the stainless-steel tube is cleaned with ultrasonic waves in alcohol. The morphology of the micro-holes is captured using a digital microscope (DVM5000, Leica, Wetzlar, Germany), and the diameters are measured with an optical microscope (SMT7-SFA, Olympus, Tokyo, Japan). Hole diameters are measured for each hole to calculate the average diameter and standard deviation.

## 4. Results and Discussion

### 4.1. Influence of the Helical Electrode Rotation

**Figure 6:**
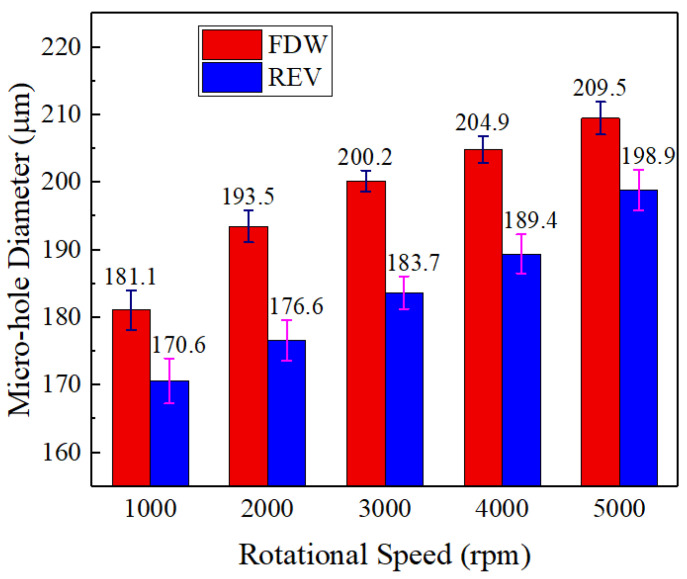
Diameter of jet holes processed by helical electrode under different rotation states.

This study investigates the influence of the rotation speed and direction of the helical electrode on the diameter of drilled jet micro-holes, with the workpiece vibration amplitude set at 8 μm and frequency at 100 Hz. The results are presented in Figure 6. Regardless of rotation direction, an increase in the rotation speed of the helical electrode leads to a gradual enlargement of the hole diameter. As indicated by the flow field simulation results (Figure 3), when the helical electrode rotates, the helical groove structure on its surface generates a stirring effect on the electrolyte within the machining gap, enhancing the flow of the electrolyte. This facilitates the rapid removal of bubbles and insoluble electrolytic products generated by the electrochemical reaction from the machining area. Additionally, as the rotational speed increases, the stirring effect of the helical electrode on the electrolyte becomes more significant. The removal rate of bubbles and insoluble electrolytic products from the micro-hole accelerates. Based on Equations (1)–(4), it can be inferred that the conductivity of the electrolyte in the machining gap is relatively high, and the removal amount of sidewall materials increases, which makes the side machining gap larger, and thus the micro-hole diameter becomes larger.

**Figure 7:**
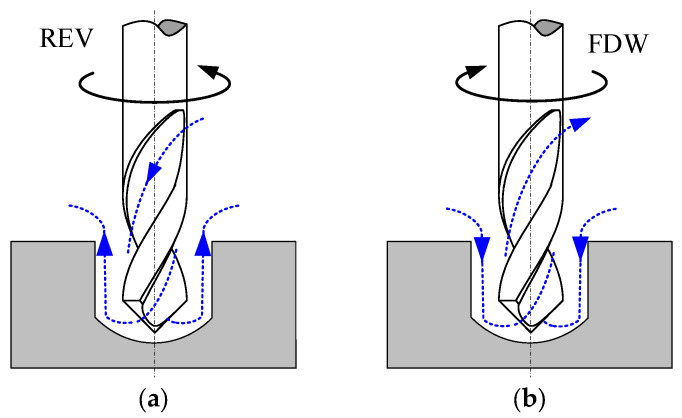
Schematic diagram of electrolyte renewal paths within the machining gap for different rotational directions of the electrode. (**a**) helical electrode rotating reversal, (**b**) helical electrode rotating forward.

For a given rotation speed, the hole diameter is larger when the helical electrode rotates in the forward direction compared to when it rotates in reverse. This is because, during the actual machining process, bubbles and insoluble electrolytic products are generated. These exist in the electrolyte and affect its conductivity, particularly the bubbles. The bubbles are produced on the surface of the helical electrode. In reverse rotation, the axial force exerted on the electrolyte is downward, with fresh electrolyte entering the hole along the electrode wall and exiting the machining gap after reaching the hole’s bottom (Figure 7a). In this flow pattern, hydrogen bubbles from the electrode wall are carried into the flow by the fresh electrolyte, reducing the electrolyte’s conductivity and resulting in a smaller hole diameter. In contrast, when rotating forward, the axial force is upward, causing spent electrolytes to be extracted along the electrode wall while fresh electrolyte enters the hole, maintaining the electrolytic reaction at the bottom of the hole (Figure 7b). This flow pattern facilitates the removal of hydrogen bubbles, wherein the bubbles precipitated from the wall of the helical electrode are directly carried out of the small hole by the electrolyte along its wall, resulting in fewer hydrogen bubbles in the electrolyte throughout the entire flow process, improving electrolyte conductivity and leading to a larger hole diameter.

### 4.2. Influence of Workpiece Vibration Amplitude

**Figure 8:**
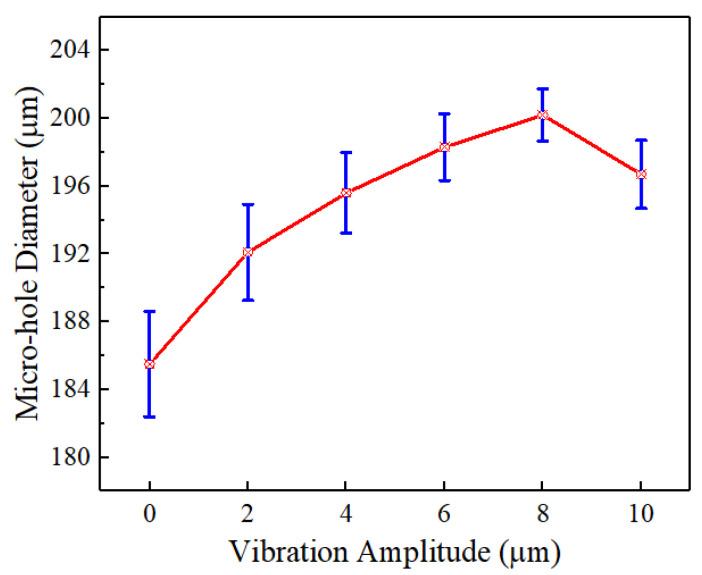
The diameter of the jet hole processed under different vibration amplitudes.

With the helical electrode rotating forward at 3000 rpm and the workpiece vibrating at 100 Hz, Figure 8 shows hole diameters for varying vibration amplitudes. At zero vibration (amplitude = 0), the smallest hole diameter and the highest standard deviation are observed. This is because, during the electrochemical drilling process, the feed rate of the helical electrode is the same as the material removal rate at the bottom of the micro-hole. As a result, the end-face machining gap between the end face of the helical electrode and the bottom of the micro-hole remains constant. The electrolyte remains relatively stationary, causing slower removal of internal bubbles and insoluble electrolytic products. This leads to a relatively low electrolyte conductivity. According to Equations (2)–(4), a smaller side machining gap results in a smaller diameter of the drilled hole. Meanwhile, the accumulation of a large number of electrolytic products within the end-face machining gap can easily cause variations in the electrolyte conductivity, leading to reduced aperture consistency and larger deviation values of micro-hole diameter.

When the workpiece vibrates upward and downward, the end-face machining gap between the end face of the helical electrode and the bottom of the micro-hole changes periodically. Based on the simulation results (Figure 4), it can be observed that when the workpiece vibrates upward, the end-face machining gap decreases, exerting a compressive effect on the electrolyte. Conversely, when the workpiece vibrates downward, the end-face machining gap increases, creating a suction effect on the electrolyte. The alternating occurrence of these two effects generates localized flow in the electrolyte, which facilitates the removal of electrolytic products within the machining gap. This results in a relatively high electrolyte conductivity and larger micro-hole diameters. Furthermore, due to the timely renewal of the electrolyte, the conductivity remains relatively constant, leading to better consistency in the micro-hole diameters produced.

As vibration amplitude increases, the hole diameter increases. This is because when the vibration frequency remains constant, an increase in vibration amplitude accelerates the movement speed of the workpiece, intensifying the compression and suction effects on the electrolyte within the end-face machining gap. This enhances the removal efficiency of electrolytic products. Consequently, the conductivity of the electrolyte increases relatively, leading to an increase in the amount of electrolytic removal and resulting in larger micro-hole diameters. However, beyond an amplitude of 10 μm, the hole diameter decreases. This is due to excessive vibration reducing the time the electrode is in close contact with the hole bottom, limiting the electrolytic reaction duration and thus reducing material removal and hole size.

### 4.3. Influence of Workpiece Vibration Frequency

**Figure 9:**
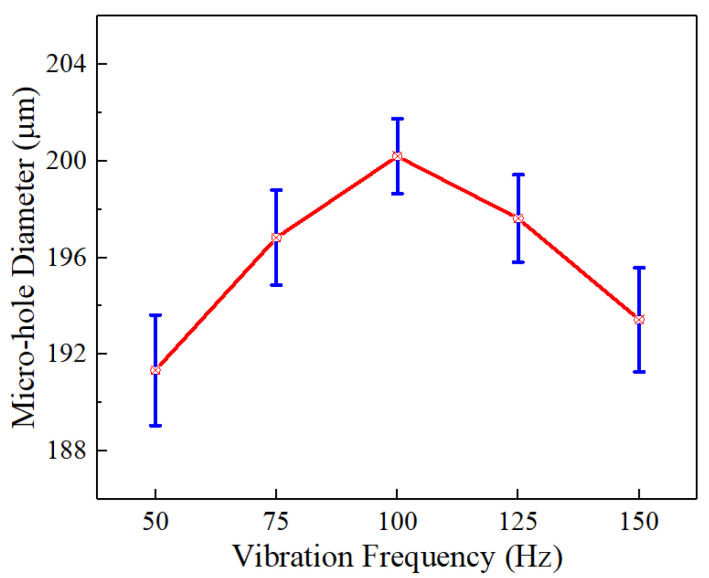
The diameter of the jet hole processed under different vibration frequencies.

When the helical electrode rotates forward at 3000 rpm, the workpiece vibrates with an amplitude of 8 μm, and Figure 9 presents hole diameters at varying vibration frequencies during the workpiece’s vibration. As frequency increases from 50 Hz to 100 Hz, the hole diameter increases from 190 μm to 200 μm. This is because when the vibration amplitude remains constant, an increase in vibration frequency leads to an increase in the number of vibrations of the workpiece per unit time, which accelerates its movement speed. This, in turn, enhances the compression and suction effects on the electrolyte within the end-face machining gap. As a result, the renewal efficiency of the electrolyte in the machining gap is improved, allowing electrolytic products to be quickly removed. This increases the conductivity of the electrolyte and results in larger micro-hole diameters being produced.

However, when the vibration frequency increases from 100 Hz to 150 Hz, the machined jet micro-hole diameter decreases from 200 μm to 185 μm. This is because the number of vibrations of the workpiece per unit of time increases when the vibration frequency increases, and the vibration time of a single time is shortened, causing the workpiece to vibrate too quickly. This results in insufficient time for the diffusion of the electrolytic products in the machining gap, hindering the renewal of the electrolyte. Therefore, the machined jet hole diameter decreases, and the consistency of the hole diameter is poor.

### 4.4. Fabrication of an Array of Jet Micro-Holes on the Wall of a Stainless-Steel Tube

**Figure 10:**
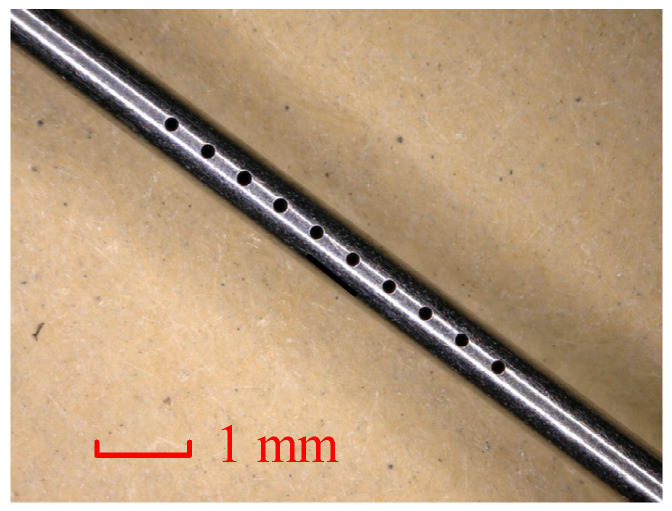
A jet micro-hole array prepared on the wall of a stainless-steel tube.

Based on the research outlined above, the helical electrode was set to rotate forward at 3000 rpm. The workpiece vibration amplitude and frequency were set to 8 μm and 100 Hz, respectively. Other processing parameters are listed in Table 2. A total of 10 arrayed jet holes, spaced 0.5 mm apart, were machined into the side wall of the stainless-steel tube, as shown in Figure 10. The average hole diameter was 200 μm, with a standard deviation of 2.97 μm.

### 4.5. Application of Tube Electrode with a Jet Micro-Hole Array in Electrochemical Cutting

**Figure 11:**
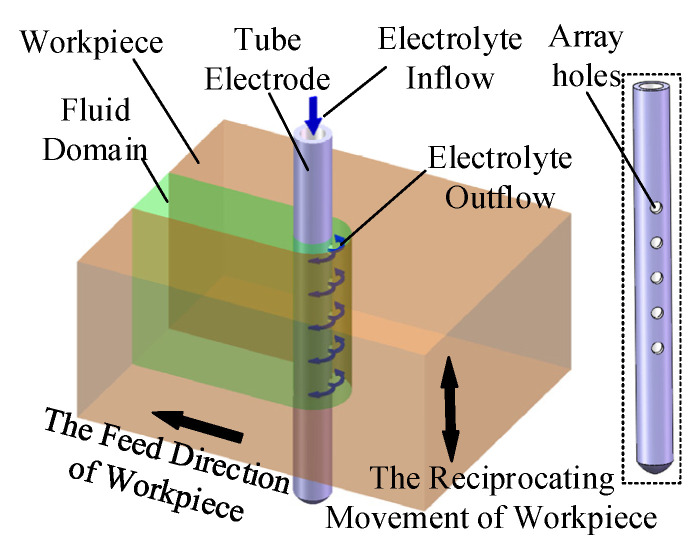
Schematic diagram of electrochemical cutting with radial electrolyte flushing [7].

**Figure 12:**
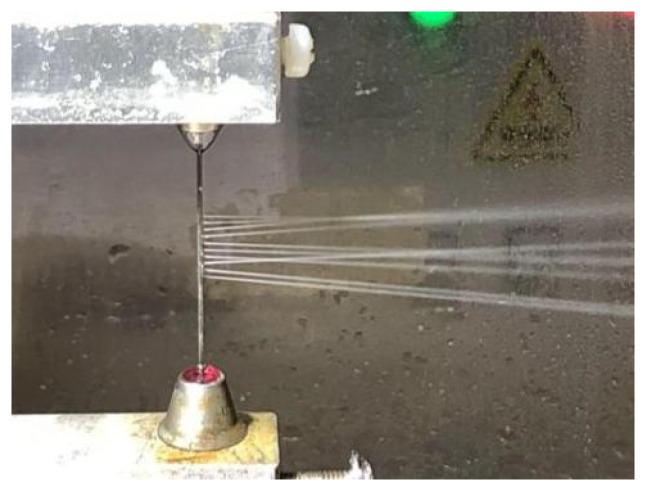
The spraying state of the electrolyte from the array of jet micro-holes in the tube wall.

**Figure 13:**
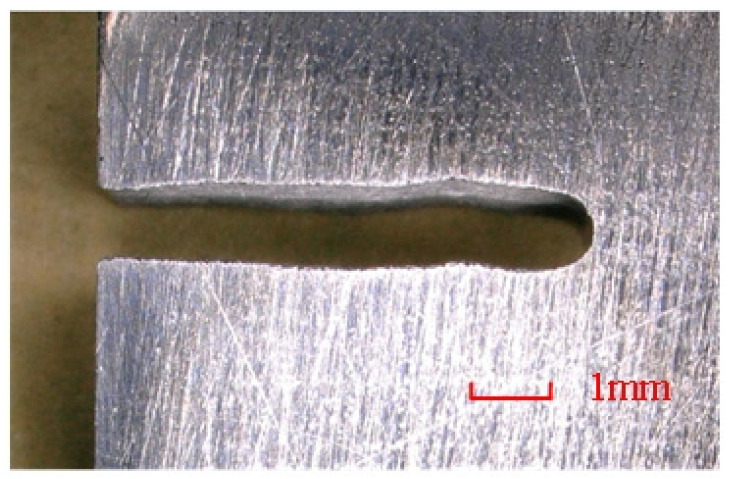
The slit structure produced by electrochemical cutting using this metal tube with jet micro-hole arrays on tube walls.

This metal tube, featuring arrayed jet micro-holes on its sidewall, was used as the tool electrode for radial electrolyte flushing electrochemical cutting experiments on a 5 mm thick stainless-steel 304 plate. The machining principle is illustrated in Figure 11, and the spraying behavior of the electrolyte from the array of jet micro-holes is depicted in Figure 12. To enhance the uniformity and consistency of the electrolyte flow within the machining gap, the workpiece underwent reciprocal motion along the tube electrode’s axial direction. The amplitude of this motion was 0.5 mm with a frequency of 1.5 Hz. Additional machining parameters are provided in Table 3. A slit structure with an average width of 1.089 mm and a standard deviation of 35.3 μm was successfully machined, as shown in Figure 13.

## 5. Conclusions

To achieve efficient and high-quality processing of jet micro-holes on the sidewall of metal tubes, a method combining helical electrode electrochemical drilling with anode vibration has been proposed. By simulating the electrolyte flow field within the machining gap and conducting machining experiments, the effects of the helical electrode’s rotation direction and speed, as well as the vibration amplitude and frequency of the workpiece, on the jet micro-hole diameter were analyzed. The following conclusions were drawn:

(1) High-speed rotation of the helical electrode and vibration of the workpiece enhance electrolyte flow within the machining gap, promoting rapid removal of electrolytic products and efficient renewal of the electrolyte;
(2) Forward rotation of the helical electrode results in larger jet micro-hole diameters compared to reverse rotation. Moreover, as the rotation speed increases, the diameter of the machined jet micro-holes also increases;
(3) Workpiece vibration leads to larger jet micro-hole diameters compared to non-vibrated processing. At optimal vibration amplitudes and frequencies, the jet micro-holes exhibit larger diameters and smaller deviations;
(4) Using the optimal machining parameters, 10 jet micro-holes were successfully machined on the stainless-steel tube wall. A radial electrolyte flushing electrochemical cutting experiment was conducted using this tube with jet micro-holes as the electrode, resulting in the formation of a slit structure on a 5 mm thick stainless-steel 304 plate.

## Figures and Tables

**Table 1:** Simulation parameters.

Parameter	Value
Diameter of helical electrode (mm)	0.1
Rotational speed (rpm)	0, 3000, −3000
Vibration amplitude (μm)	10
Vibration frequency (Hz)	100
Diameter of hole (mm)	0.2
Depth of hole (mm)	0.1
End-face machining gap (μm)	20

**Table 2:** Experimental parameters.

Parameter	Value
Stainless-steel tube	Outer diameter 0.7 mm Inner diameter 0.4 mm
Helical electrode	Diameter 0.1 mm Screw pitch 0.48 mm
Electrolyte	NaNO_3_ solution 50 g/L
Electrical parameter	8 V-25%-100 kHz
Feed rate (μm/s)	0.8
Feed quantity (μm)	200
Helical electrode rotation speed (rpm)	1000, 2000, 3000, 4000, 5000
Workpiece vibration amplitude (μm)	0, 2, 4, 6, 8, 10
Workpiece vibration frequency (Hz)	50, 75, 100, 125, 150

**Table 3:** Machining parameters of electrochemical cutting with radial electrolyte flushing.

Parameter	Value
Electrical parameter	12 V-35%-50 kHz
Electrolyte type	NaNO_3_ solution
Electrolyte concentration (g/L)	100
Inlet pressure (MPa)	2.0
Feed rate (μm/s)	4

## Data Availability

The original contributions presented in this study are included in the article. Further inquiries can be directed to the corresponding author.
